# Enhancing Mushroom Freezing Quality Using Microwave-Assisted Technology

**DOI:** 10.3390/foods13172805

**Published:** 2024-09-03

**Authors:** Majid Yousefi Vardanjani, Nasser Hamdami, Mohsen Dalvi-Isfahan, Alain Le-Bail

**Affiliations:** 1 Department of Food Science and Technology, College of Agriculture, Isfahan University of Technology, Isfahan 84156-83111, Iran; 2 ONIRIS—GEPEA (UMR CNRS 6144), Site de la Géraudière CS 82225, CEDEX 3, 44322 Nantes, France; 3 Department of Food Science and Technology, Faculty of Agriculture, Jahrom University, Jahrom 74137-66171, Iran

**Keywords:** microwave-assisted freezing (MAF), mushrooms, quality attributes, energy consumption

## Abstract

This study investigated the effects of microwave-assisted freezing on the quality attributes of button mushrooms (*Agaricus bisporus*). Four levels of microwave power (0, 10, 20, 30%) were applied to the mushroom samples during freezing. The quality attributes of the frozen and thawed mushrooms were then evaluated. The results suggested that higher microwave power produced the smaller and more uniform ice crystals. Moreover, the browning index of the mushroom samples increased with increasing microwave power. The textural properties (hardness) of the mushrooms were also affected by the microwave power, showing higher values as the power increased. Furthermore, the ratio of the microwave operating system’s power to the freezer power was low and approximately 20% at the highest power level. Therefore, these findings confirm the potential of microwave-assisted freezing for reducing freeze damage to mushroom tissue and, thus, provide frozen mushroom with a better texture.

## 1. Introduction

Freezing is a preferred method for preserving food, especially for maintaining freshness. However, plant-based food presents unique challenges during freezing due to its high water content and the varying shapes and sizes, which strongly affect the heat transfer and freezing rate [1,2]. Novel freezing technologies are being developed to mitigate damage in the freezing process of plant-based foods. These technologies can be broadly classified into three categories: (i) those that increase heat transfer speed during freezing, (ii) those that alter food material characteristics, and (iii) adjunct technologies that help control ice formation. The aim of these technologies is to manipulate ice crystal properties or alter water and ice thermodynamics, kinetics, and structure [3,4]. Electric and magnetic fields can be used to manipulate the freezing process of water by controlling ice crystal nucleation and growth. The characteristics of these fields can influence the shape, size, and freezing rate of the ice crystals. Some examples of these fields include static and alternating electric fields, static and oscillating magnetic fields, and electromagnetic radiation (EMR) [5,6,7,8,9].

Microwave-assisted freezing (MAF) is one of the EMR methods of freezing that has been studied by a few researchers [10,11]. Results showed that freezing in the presence of microwave radiation significantly reduced the damage to food microstructure and improved the quality of the frozen product. Graziele et al. (2020) and Jha et al. (2020) proposed two concepts regarding the mechanism of action of MAF on ice crystal size reduction, namely NITOM and NIMIW. NITOM, which stands for nucleation induced by temperature oscillation caused by microwaves, assumes that microwave radiation can create temperature fluctuations in the food matrix, which can initiate ice nucleation at higher subzero temperatures [12,13,14]. This results in smaller and more uniform ice crystals, which can enhance the quality of the frozen food. NIMIW, which stands for nucleation induced by constant or pulsed microwave power levels, assumes that microwave radiation can directly alter the molecular structure and orientation of water molecules, which can increase the nucleation rate and decrease the supercooling degree. This leads to smaller and more dispersed ice crystals, which can preserve the quality of the frozen food. However, the significance of the second concept was demonstrated in the results obtained by Sadot et al. (2020) [15]. They found that MAF reduced the ice crystal size of methylcellulose gels by up to 25% for both pulsed and continuously applied microwaves. This reduction could be attributed to the perturbation of the H-bond network in water structures during freezing [15].

The button mushroom, a popular food, spoils quickly after harvest due to various factors, such as high respiration rate, high activity of browning enzymes, water loss, and microbial attack. While most research has focused on post-harvest treatments and packaging to increase shelf life [16], some studies have found that freezing under an electric field or ultrasound irradiation can improve the mushroom’s properties [17,18].

Previous studies have documented the application of MAF in plant food commodities such as potatoes and apples; however, investigations into its use for more delicate plant-based products, like button mushrooms, remain absent from the literature. Due to their considerable water content, tender structure, and pronounced enzymatic activity, button mushrooms exhibit a heightened sensitivity to freezing processes when compared to potatoes and apples. Hence, this study aims to explore the effects of microwave radiation on the quality of frozen mushrooms, including aspects like ice crystal size, texture, color, and energy consumption. The findings could contribute to the development of an efficient microwave-assisted freezing device for large-scale processing.

## 2. Materials and Methods

### 2.1. Materials

The button mushrooms used in this research were obtained fresh and daily. The mushrooms were transported to the laboratory within 1 h after picking, then stored in darkness at 4 ± 1 °C and 90% RH. Mushrooms with appropriate and uniform size, color, and texture were selected. To ensure consistency, the mushrooms were separated based on cap color and size. Specifically, mushrooms with a cap size ranging from 5 to 7 cm and a white color without any dark spots were chosen. Next, mushroom samples were prepared by cutting them using a cylindrical blade. The samples were cut into cylindrical pieces with a diameter of 14 mm and a height of 10 mm, with an average weight of 1.0 ± 0.11 g. The materials used in this study were of analytical grade with a purity of 99.9%, sourced from Sigma (St. Louis, MO, USA) and Merck (Darmstadt, Germany).

### 2.2. Microwave Freezing System

**Figure 1:**
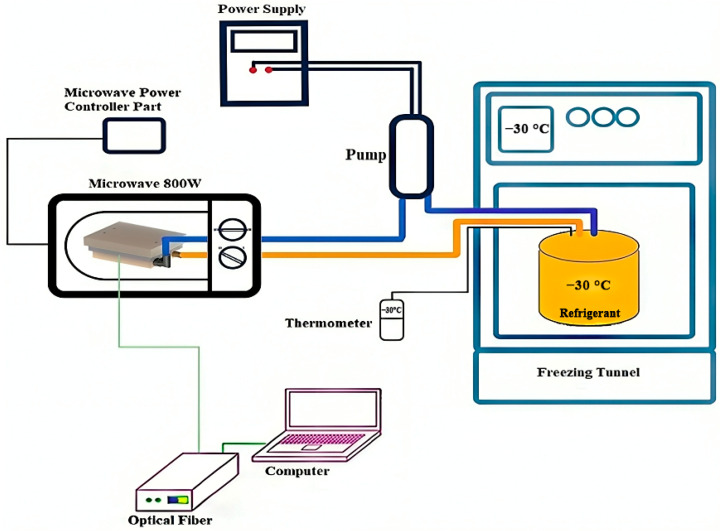
Schematic diagram of the experimental set-up.

**Figure 2:**
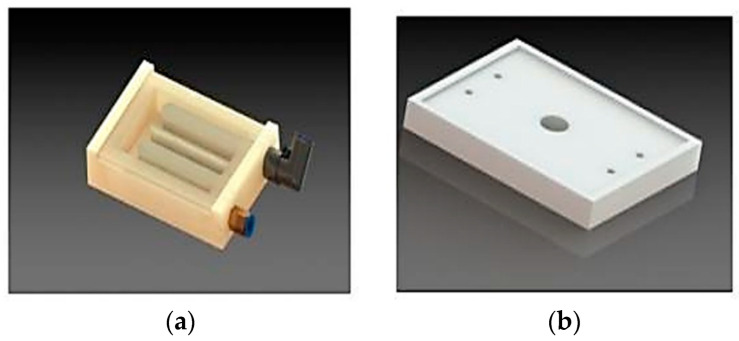
Diagram of the various components of the experimental setup, including (**a**) the heat exchanger and (**b**) the sample holder.

The microwave-assisted freezing system used in this study was similar to the one described by Atani et al. (2022) [10]. A schematic diagram of the setup is presented in Figure 1. The system comprised a multimode domestic microwave oven (Samsung M1719N, 800 W, 2.45 GHz, Budapest, Hungary), a device for controlling microwave power at the lower oven mode (100 W), a heat exchanger (Figure 2a), and a sample holder constructed from polystyrene foam (Figure 2b).

The sample and the refrigerant (ethanol) were separated by a tempered glass plate, 2.5 mm thick, that was placed on the top of the heat exchanger made of an Ertalon^®^ block. The heat exchanger was connected to a cooling bath by plastic tubes that had an inlet and an outlet. The cooling bath was in a freezing tunnel with a temperature of −30 °C, and the refrigerant circulated in the plastic tubes at 3 L/min using an external diaphragm pump. The tubes were insulated to prevent heat loss. The heat exchanger and the sample holder were in the middle of the microwave oven. The temperature of the sample was recorded every second and monitored by an optical fiber during freezing (FOB100 Series, OMEGA Engineering, Inc., Richmond Hill, ON, Canada).

### 2.3. Microwave-Assisted Freezing (MAF)

Mushroom samples were placed in a cylindrical cell within the sample holder. The optical fiber’s tip was positioned at the geometric center of the sample, at mid-height. The samples were then subjected to controlled cooling. Microwave power was regulated by a power level controller that modulated the microwave radiation in short intervals. This study examined the impact of varying microwave power levels on the microwave-assisted freezing (MAF) of mushrooms. Power levels were controlled via duty cycles (on time/total time), with 0% duty cycle (no microwave exposure) serving as the control group. Treatment groups utilized 10%, 20%, and 30% duty cycles, corresponding to 3, 6, and 9 s of microwave exposure per 30 s cycle. Duty cycles above 30% were avoided to prevent overheating of the refrigerant. Temperature at the mushroom’s center was recorded during freezing using the optical fiber, connected to a temperature measurement system (FOB100 Series, 4-channel, accuracy ± 0.8 °C, OMEGA Engineering, Inc., Richmond Hill, ON, Canada). Data were logged at one-second intervals using FOB100 Windows software, Version 1 [10].

### 2.4. Microstructural Analysis

The protocol suggested by Fallah-Joshaqani et al. (2021) was followed to examine the microstructure of mushroom samples using the light microscopy method [17]. After freezing, the frozen mushroom samples were immersed in Carnoy’s solution (ethanol: acetic acid: chloroform; 0.6: 0.3, 0.1 *v*/*v*) for fixation. The samples were then dehydrated by increasing the ethanol concentration (50%, 70%, 90%, 100%). Next, the tissues were immersed in xylene solution and then kept in liquid paraffin for 1 h. Using a microtome, the samples were sectioned into 5 μm thick slices after embedding them in paraffin blocks (RM-2055, Leica Microsystems, Wetzlar, Germany). The sections were placed on glass slides and heated in an oven at 56–58 °C (the melting point of paraffin). The slides were stained with Rose Bengal dye solution for 2 min and observed with a light microscope at a magnification of 400 to 100× (Olympus BX41, Olympus Optical Co. Ltd., Tokyo, Japan). Two images per sample were taken using an Olympus DP12 CCD digital camera (Olympus Optical Co. Ltd., Tokyo, Japan).

The image analysis was performed on ImageJ software (version 1.52a, Java 1.8.0_112, Wayne Rasband, National Institutes of Health, Bethesda, MD, USA). The images were first calibrated to change the image scale to metric units. Preprocessing was conducted to eliminate issues caused by lack of contrast, presence of noise, and non-uniform illumination in acquired images. Otsu’s thresholding segmentation method was applied to separate the tissue (purple) from the background (white) in the image. The median filter was then used to remove floating pixels. Subsequent object counting and measurement of the morphological characteristics of ice crystals was conducted.

Three morphological parameters were measured to assess the effects of MAF on the microstructures of mushroom samples: equivalent diameter, total relative ice area, and fractal dimensions (FD) [19]. The diameter of a circle that matches the area of the object is called the equivalent diameter. The total relative ice area is the fraction of the object area that is occupied by the ice crystals at a certain point. Fractal dimension was calculated by the FracLac plugin in the ImageJ software, developed by Karperien (2012) [20]. According to the definition, the fractal dimension varies from 1 to 2 and a fractal value close to 1 represents that the boundary of the object under analysis has smooth boundaries, while a value close to 2 represents a high degree of tortuosity [21].

### 2.5. Drip Loss Determination

The frozen sample’s weight was measured before and after thawing at +4 °C for 2.5 h using an analytical balance with an accuracy of +0.001 g and the drip loss percentage was computed using Equation (1).

(1) $$\mathrm{Drip}\;\mathrm{loss}\;\left(\%\right)=\frac{{(M}_{1}-{\;M}_{2})}{M_{1}}\times 100\;$$

where M_1_ and M_2_ are the weight of frozen and thawed samples, respectively.

### 2.6. Texture Profile Analysis

In order to evaluate the effect of freezing mushroom samples under different microwave powers, the texture profile analysis (TPA) was conducted using a Texture Analyzer (SANTAM, Tehran, Iran). The compression of the mushroom sample was performed by using an aluminum cylinder probe (45 mm diameter) at two separate stages, such that the loading speed was 1 mm/s, the delay time between the two compression stages was 5 s, and the compression of the mushroom sample was considered to be 50% of the initial thickness. The textural parameters of hardness (N) were calculated from the texture profile data [22].

### 2.7. Color Analysis

A colorimetric device (ZE-6000 Nippon Denshoku, Kogyo Co., Tokyo, Japan) was used to examine the surface color of mushroom samples after thawing. The device was calibrated with a white plate (L* = 95.26, a* = −0.89, b* = 1.18) as a reference. The color values were obtained by averaging three measurements from different faces of the mushroom surface. The color parameters of lightness (L*), redness (a*), and yellowness (b*) were determined for each sample. The total color change between fresh and thawed samples was computed using Equation (2) [22].

(2) $$\Delta \;E=\sqrt{{\left(L^{*}-L\right)}^{\;2}+{\left(a^{*}-\;a\right)}^{\;2}+{\left(b^{*}-b\right)}^{\;2}}$$

where L*, a*, and b* are the color values of thawed samples and L, a, and b are the color values of fresh samples. The browning index (BI) and total whiteness (WI) of the samples were calculated using Equations (3) and (4), respectively [16].

(3) $$\mathrm{BI}=581.395\left\{\left(\frac{a^{*}+1.75L^{*}}{5.645L^{*}+a-3.012b^{*}}\right)-0\cdot 31\right\}$$

(4) $$\mathrm{WI}=100-\sqrt{\left[{\left(100-L^{*}\right)}^{\;2}+{a^{*}}^{\;2}+{b^{*}}^{\;2}\right]}$$

### 2.8. Energy Consumption

The energy consumption of the microwave oven, the microwave power level controller, and circulating pump were measured during MAF under various microwave powers and impulse durations. A power analyzer (DW-6090A Lutron Electronic Enterprise Co., Ltd., Taipei, Taiwan) was used to measure power, while voltage and current were measured with a clamp meter and a digital true RMS multi-display multimeter, respectively.

### 2.9. Statistical Analysis

The tables and figures in this study showed the mean value ± standard error s. Drip loss, texture, microstructure, and color analyses were conducted in triplicate for each treatment level. Additionally, freezing time was measured using ten independent samples per selected microwave power level. Data analyses were performed using the SPSS software (version 23.0; SPSS, Chicago, IL, USA). One-way ANOVA was used to analyze the data. The LSD test was employed to identify the mean significance differences among the treatments. A significance level of 0.05 was chosen for the analysis.

## 3. Results and Discussion

### 3.1. Temperature Profile and Freezing Rate

**Figure 3:**
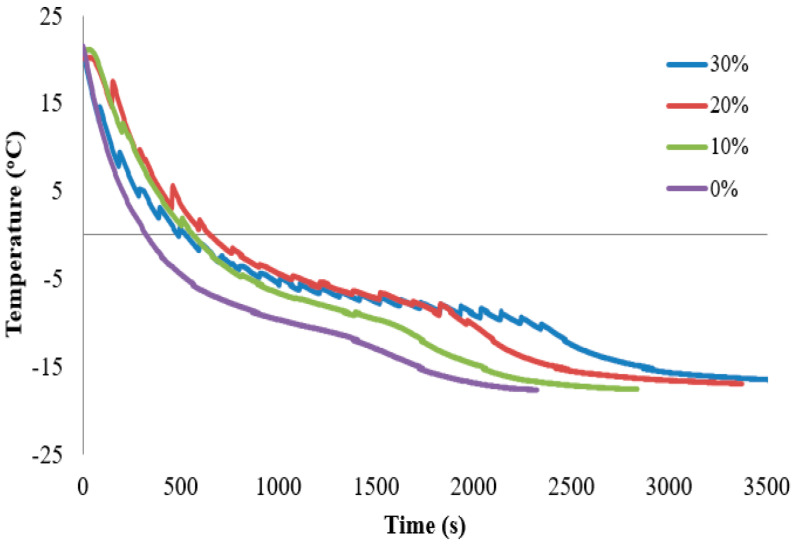
The real-time temperature profile of mushroom samples as a function of different power levels of microwave irradiation during freezing.

The real-time temperature profile of mushroom freezing under different levels of pulsed microwave irradiation is shown in Figure 3. The control sample, which was frozen without any microwave irradiation, had a smooth temperature curve. However, for the samples that were exposed to microwave irradiation, temperature fluctuations were evident due to the microwave pulses [13]. In other words, the mushroom sample undergoes a process of heat generation and heat removal when the microwave is on. The pulsed microwave radiation is absorbed by the sample and converted into heat energy. At the same time, the heat exchanger transfers heat from the sample to the refrigerant. This dual process of microwave heating and heat exchanger cooling results in the sample solidifying through a decreasing temperature that oscillates. The microwave power affects the rate of temperature oscillation, which increases as the power increases [10]. The observation is in agreement with the NITOM concept, where fluctuating temperature induced by pulsed microwave energy impacts secondary nucleation [13].

Freezing time is recognized as one of the most important factors affecting final product quality in frozen foods. Freezing time was defined as the time required to change the temperature from −1 °C (initial freezing temperature of the mushroom) to −7 °C (80% of total water is converted to ice) at a center point in the system [1]. The results show that statistically significant differences existed between the freezing time of the samples. In general, the total freezing time in microwave irradiated samples was longer compared to the control group (Table 1).

Moreover, the effects of different power levels of microwave irradiation on the freezing rates were examined within the temperature range of −5 °C to −10 °C. The freezing rates for various microwave power levels during the freezing process are shown in this temperature range (Table 1). The statistical analysis revealed a significant difference between the freezing rates of the conventional and the microwave-assisted freezing methods, with the latter having lower rates. These findings are consistent with previous studies that reported a decrease in the freezing rate as the power level increased [11].

### 3.2. Microstructure Analysis

**Figure 4:**
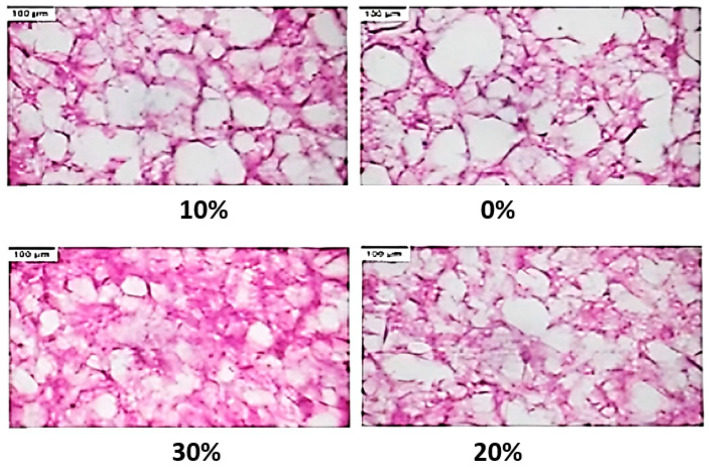
Micrograph of button mushroom frozen by applying different levels of microwave power (0, 10, 20, and 30 percent). Scale Bar = 100 μm.

The developed microwave-assisted freezing process was applied to the mushroom samples and their microstructure was examined (Figure 4). The tissue of mushroom is shown by the purple color and the white zones are the voids created by the formation of ice crystals. The area of the white zone reduces as the power level of microwave irradiation increases. This implies that the control sample had more structural damage. Images were analyzed using ImageJ software to measure morphological parameters and assess the effect of MAF on the ice crystals’ morphology (Table 2). While the equivalent diameter of ice crystals and the total relative ice area decreased significantly with increasing microwave power levels, the largest ice crystals were observed in the control group. The average ice crystal size in the control samples was 52.8 micron, which decreased to 38.3 micron under 30% power level.

The results from the current study support the general findings from other similar studies, where MAF had a significant effect on ice crystal morphology. For example, Xanthakis et al. (2014) reported that the equivalent diameter of ice crystals in pork meat reduced by 62% at a microwave power level of 60% [11]. Jha et al. (2019) also reported that the MAF process produced a better microstructure than the control condition in plant tissue [13]. In a study conducted by Sadot et al. (2020), the X-ray micro-tomography method confirmed that microwave assistance during freezing significantly reduces the size of the ice crystal for both pulsed and continuous radiation. Their results also indicated that temperature fluctuations caused by microwave radiation did not play a significant role in reduction of the size of ice crystals [14]. The reduction in the size of ice crystals due to microwaves can be explained based on the NIMIW concept, where microwaves exert a torque on water molecules, disrupting their equilibrium relationships within ice clusters. As a result, existing ice crystals in the form of nuclei fragments, creating new nucleation sites. This process promotes secondary nucleation and ultimately leads to a reduction in ice crystal size [13].

Microstructure analysis showed that the ice crystals in the control group had the lowest value of fractal dimension. These results are in agreement with findings of Dalvi-Isfahan et al. (2016), who observed an increase in fractal dimensions of lamb meat with increasing intensity of the electrostatic field [19]. Thus, the results of both studies indicate that freezing combined with electrical disturbances decreased the smoothness of the surface of ice crystals. The importance of ice crystal shape on product quality was reported by Petzold and Aguilera (2009), who found that round and smooth ice crystals are able to give a smooth body and texture to the frozen product [23]. The findings of this study highlight that microwave irradiation can produce finer ice crystals that can enhance the quality of the product.

### 3.3. Drip Loss

**Figure 5:**
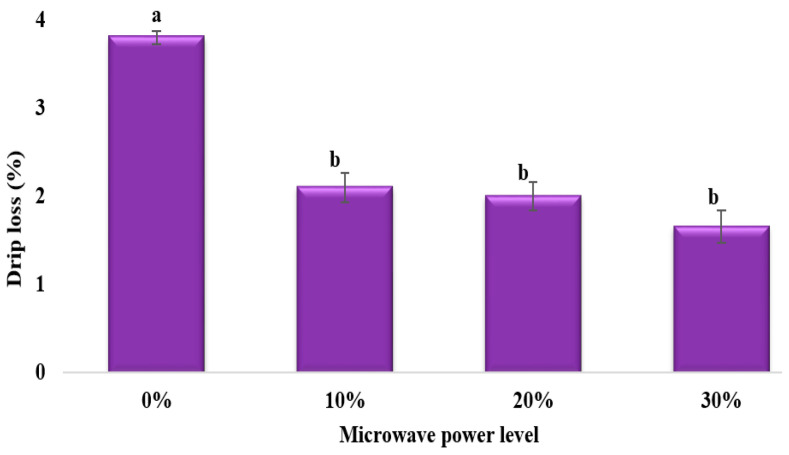
Effects of different power levels of MAF on drip loss of freeze-thawed mushroom samples. Values with the same letter are not statistically different at the 0.05 level according to the LSD test.

Bevilacqua et al. (1979) showed that the injury induced by freezing tissue can be determined by measuring the volume of drip produced during thawing [24]. The amount of drip loss released by frozen mushroom samples under various levels of microwave power is shown in Figure 5. The control sample had the most drip loss, and the drip amount reduced significantly (*p* < 0.05) as the microwave power increased. The microstructural images reveal the reason for the different drip loss levels during thawing. The ice crystals formed during freezing were smaller with microwave assistance, which caused less cellular damage, and this was reflected by a lower drip loss amount. These findings are also in accord with results obtained for potatoes by Jha et al. (2020) [13].

### 3.4. Hardness

**Figure 6:**
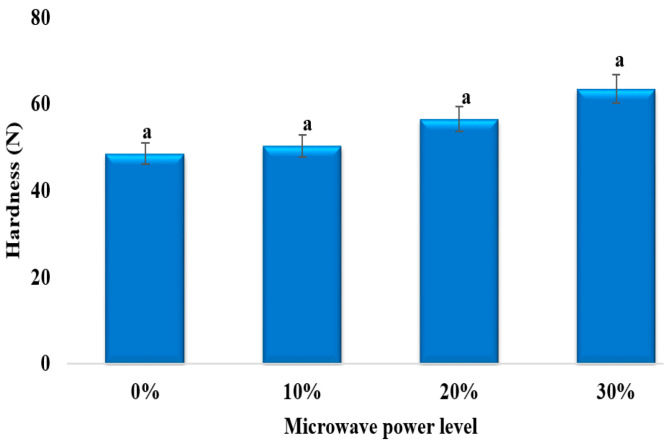
Effects of different power levels of MAF on hardness value of freeze-thawed mushroom samples. Values with the same letter are not statistically different at the 0.05 level according to the LSD test.

Figure 6 compares the hardness of mushroom samples under different microwave power levels. The hardness of fresh mushrooms decreased significantly after freezing, where hardness was best retained by microwave irradiation at higher power levels. Hardness generally depends on the crystal size in a system, with smaller and more abundant crystals usually resulting in a harder product. Previous studies from Fallah-Joshaqani et al. (2021) and Islam et al. (2014) have shown that mushroom hardness improved due to the decreased ice crystal size caused by ultrasound-assisted freezing and high-voltage electric-field-assisted freezing, respectively [17,22]. This is also consistent with Jha et al. (2020), who found that potato samples frozen under MAF maintained their firmness better than the control sample [13]. Similarly, the present study demonstrates that microwave radiation during freezing reduced the ice crystal size in mushrooms, which minimized the damage to the cell structure and, consequently, increased the hardness.

### 3.5. Color Evaluation

Table 3 shows the total color difference, lightness index, and browning index calculated between the thawed and fresh samples. Mushroom samples that experienced microwave power levels of 10% had the lowest value of total color difference after thawing, while samples frozen at power levels of 20% and 30% showed the highest differences. Different levels of microwave power after thawing did not result in a significant difference in the browning and whiteness index (*p* > 0.05). However, the browning index rose and the whiteness index fell as the microwave power levels increased. This may be due to the activity of peroxidases (POD) and polyphenol oxidase (PPO) enzymes, which are responsible for the browning of mushrooms during the freezing process [25,26]. Table 1 indicates that the freezing time increased considerably with higher microwave power levels, providing more chances for browning enzymes to darken the tissue. Since longer freezing times provide more opportunities for browning enzymes to darken the tissue, higher microwave power levels also increase the chances for browning enzymes to darken the tissue. To decrease this defect, strategies such as the utilization of antioxidants and chelating agents in the freezing process can effectively reduce browning and maintain the quality of mushroom products.

### 3.6. Energy Consumption

**Figure 7:**
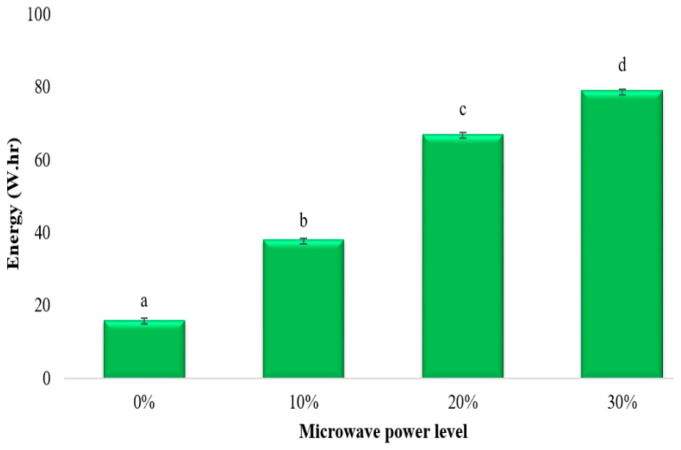
Effects of different power levels of MAF on energy. Values with the same letter are not statistically different at the 0.05 level according to the LSD test.

The electric power measurement system measured the energy consumed by the microwave device, the diaphragm pump, and the controller part. The power analyzer installed on the three-phase circuit supplying the motor measured the energy use of the freezer, which was about 595 W. The findings of this study showed that the freezer used up most of the energy, and the proportion of the power of the microwave operating system to the freezer power was around 20% at the maximum power level. In other words, the energy demand of the freezer significantly influenced the energy use. Moreover, the power consumption was influenced by the microwave power levels, and it increased as the power increased (Figure 7).

## 4. Conclusions

The study investigated the effects of MAF on the quality and energy consumption of mushroom samples. The results showed that MAF increased the freezing time and decreased the freezing rate compared to the conventional freezing method. The MAF power levels affected the quality parameters of the frozen mushrooms. Higher power levels reduced the ice crystal size and the drip loss, and increased the hardness, but they also made the browning index higher. This might be because the freezing time was longer at higher power levels, which allowed more browning enzymes to work. The energy consumption of the freezing system was mainly determined by the freezer, and it increased with the microwave power levels. Therefore, MAF can be considered as a potential alternative to enhance the quality of frozen mushrooms, but the optimal power level should be selected to maintain the quality.

## Figures and Tables

**Table 1:** Freezing time under different microwave power levels *.

Microwave Power Level	0%	10%	20%	30%
Freezing time (min)	19 ± 1.1 ^a^	26 ± 1.6 ^b^	33 ± 2.1 ^c^	39 ± 2.4 ^d^
Freezing rate (Δ°C/min)	1.72 ± 0.22 ^a^	1.19 ± 0.2 ^ab^	1.03 ± 0.19 ^bc^	0.76 ± 0.25 ^c^

* Data represent average values over 10 replicates per treatment. Different letters in each row indicate significant difference (*p* < 0.05).

**Table 2:** Effects of different power levels of MAF on ice crystal properties of freeze-thawed mushroom samples.

Microwave Power Levels (%)	0%	10%	20%	30%
Total relative ice area (%)	58.5 ± 3.1 ^a^	49.3 ± 2.5 ^b^	47.1 ± 2.6 ^bc^	44.8 ± 3.3 ^c^
Equivalent diameter (µm)	52.8 ± 3.42 ^a^	45.9 ± 1.56 ^ab^	40.61 ± 1.31 ^b^	38.3 ± 3.86 ^b^
Fractal dimensions (1–2)	1.84 ± 0.004 ^a^	1.86 ± 0.004 ^b^	1.88 ± 0.002 ^c^	1.89 ± 0.006 ^c^

Different letters in each row indicate significant difference (*p* < 0.05).

**Table 3:** Effects of different power levels of MAF on color parameters of freeze-thawed mushroom samples.

Microwave Power Levels (%)	0%	10%	20%	30%
Browning Index	27.5 ± 2.0 ^a^	32.6 ± 3.3 ^a^	32.6 ± 2.1 ^a^	32.8 ± 2.8 ^a^
Whiteness Index	72.4 ± 2.62 ^a^	72.3 ± 4.68 ^a^	67.2 ± 2.78 ^a^	60.8 ± 4.53 ^a^
Total color difference	11.4 ± 1.71 ^ab^	7.25 ± 1.57 ^b^	13.2 ± 3.21 ^ab^	24.15 ± 3.42 ^b^

Different letters in each row indicate significant difference (*p* < 0.05).

## Data Availability

The original contributions presented in the study are included in the article, further inquiries can be directed to the corresponding author.
